# Patient Repositioning during Hospitalization and Prevention of Pressure Ulcers: a Narrative Review

**DOI:** 10.17533/udea.iee.v42n1e07

**Published:** 2024-04-28

**Authors:** Olga L. Cortés, Skarlet M. Vásquez

**Affiliations:** 1 Nurse, PhD. Associate researcher, Department of Research and Nursing. Fundación Cardioinfantil, Instituto de Cardiología, Bogotá, Colombia. Email: ocortes@lacardio.org. Corresponding author Department of Research and Nursing Fundación Cardioinfantil Instituto de Cardiología Bogotá Colombia ocortes@lacardio.org; 2 Nurse, Master’s. Associate professor, Nursing Program, Universidad Autónoma de Bucaramanga, Floridablanca, Colombia. Email: svasquez196@unab.edu.co Universidad Autónoma de Bucaramanga Nursing Program Universidad Autónoma de Bucaramanga Floridablanca Colombia svasquez196@unab.edu.co

**Keywords:** pressure ulcer, moving and lifting patients, prevention & control, nursing., úlceras por presión, prevención & control, movimiento y levantamiento de pacientes, enfermería., úlcera por pressão, movimentar e levantar pacientes, prevenção & controle, enfermagem.

## Abstract

**Objective.:**

This article presents a literature review to explore and analyze the current situation of pressure ulcers or lesions or decubitus ulcers, pathophysiological, epidemiological aspects, and risk factors. The progress in evidence of the effectiveness of preventive repositioning in the appearance of these lesions in vulnerable hospitalized patients is also evaluated.

**Methods.:**

Databases were reviewed in non-systematic manner, including the Cochrane Wounds Specialized Register; Medline, Scopus, PubMed, the Cochrane Central Register of Controlled Trials; MEDLINE (Ovid); EMBASE (Ovid), Web of Science, SciELO, and Lilacs. The general search terms included [pressure ulcers or pressure lesions or decubitus ulcers] and [prevention or preventive] and [repositioning or positioning or position changes or postural change] and [patient at risk or vulnerable] and [hospitalized or ICU or intensive care]. Systematic literature reviews, randomized clinical trials, observational studies, cost-effectiveness and qualitative studies in English or Spanish were included.

**Results.:**

Although globally, the incidence, prevalence, and years of disability associated to these lesions has diminished between 1990 and 2019, the high impact on health persists. Evidence found on the effectiveness of repositioning in preventing pressure ulcers and health associated costs has been evaluated with certainty between low and very low, as a result of conducting research with serious methodological limitations that report results with high inaccuracy.

**Conclusion.:**

The findings reported present that these lesions persist at hospital level and continue being a global social and health problem with high impact on health budgets. Likewise, there is a need to develop greater quality research on prevention strategies, such as repositioning, which validate their effectiveness, and justify their use.

## Resumen

**Objetivo.:**

Este artículo presenta una revisión de la literatura con el objetivo de explorar y analizar la situación actual de las úlceras o lesiones por presión o úlceras por decúbito, aspectos fisiopatológicos, epidemiológicos, y factores de riesgo. Se evalúa además el progreso en la evidencia de la eficacia del reposicionamiento preventivo en la aparición de estas lesiones en pacientes vulnerables hospitalizados.

**Métodos.:**

Se revisaron bases de datos de forma no sistemática, incluyendo The Cochrane Wounds Specialised Register; Medline, Scopus, PubMed, the Cochrane Central Register of Controlled Trials; MEDLINE (Ovid); EMBASE (Ovid), Web of Science, Scielo, y Lilacs. Los términos de búsqueda generales incluyeron [úlceras por presión o lesiones por presión o úlceras por decúbito] y [prevención o preventivo] y [reposicionamiento o posicionamiento o cambios de posición o cambio postural] y [paciente en riesgo o vulnerable] y [hospitalizado o UCI o cuidados intensivos]. Se incluyeron revisiones sistemáticas de la literatura, ensayos clínicos aleatorizados, estudios observacionales, estudios de costo-efectividad y cualitativos en idioma inglés o español.

**Resultados.:**

Aunque globalmente la incidencia, prevalencia y años de incapacidad asociado a estas lesiones ha disminuido entre 1990 y 2019, el impacto en salud persiste de forma elevada. La evidencia encontrada sobre la eficacia del reposicionamiento en prevención de úlceras por presión y costos asociados en salud ha sido evaluada con certeza entre baja y muy baja, como resultado de la realización de investigaciones con serias limitaciones metodológicas que reportan resultados con alta imprecisión.

**Conclusión.:**

Los hallazgos reportados presentan que estas lesiones persisten a nivel hospitalario y continúan siendo un problema social y de salud mundial con alto impacto en los presupuestos en salud. Así mismo se presenta la necesidad de desarrollar mayor investigación de calidad en estrategias preventivas como el reposicionamiento, que validen su eficacia, y justifiquen su utilización.

## Resumo

**Objetivo.:**

Este artigo apresenta uma revisão da literatura com o objetivo de explorar e analisar a situação atual das úlceras por pressão ou úlceras de decúbito, os aspectos fisiopatológicos e epidemiológicos e os fatores de risco. Também avalia o progresso na evidência da eficácia do reposicionamento preventivo no desenvolvimento dessas lesões em pacientes hospitalizados vulneráveis.

**Métodos.:**

Foram revisados bancos de dados não específicos do local, incluindo The Cochrane Wounds Specialised Register; Medline, Scopus, PubMed, Cochrane Central Register of Controlled Trials; MEDLINE (Ovid); EMBASE (Ovid), Web of Science, Scielo e Lilacs. Os termos gerais de pesquisa incluíram [úlceras de pressão ou lesões por pressão ou úlceras de pressão ou úlceras de decúbito] e [prevenção ou preventivo] e [reposicionamento ou posicionamento ou mudanças de posição ou mudança postural] e [paciente em risco ou vulnerável] e [hospitalizado ou UTI ou terapia intensiva]. Foram incluídas revisões sistemáticas da literatura, ensaios clínicos randomizados, estudos observacionais, estudos de custo-efetividade e qualitativos em inglês ou espanhol.

**Resultados.:**

Embora, em geral, a incidência, a prevalência e os anos de incapacidade associados a essas lesões tenham diminuído entre 1990 e 2019, o impacto na saúde continua alto. As evidências encontradas sobre a eficácia do reposicionamento na prevenção de úlceras por pressão e os custos de saúde associados foram avaliadas com certeza baixa a muito baixa, como resultado de pesquisas com sérias limitações metodológicas que relataram resultados altamente imprecisos.

**Conclusão.:**

Os resultados relatados mostram que essas lesões persistem em nível hospitalar e continuam a ser um problema social e de saúde global com alto impacto nos orçamentos de saúde. Também há necessidade de mais pesquisas de qualidade sobre estratégias preventivas, como o reposicionamento, para validar sua eficácia e justificar seu uso.

## Introduction

Pressure ulcers (PU), decubitus ulcers (DU) or also denominated pressure lesions (PL) are formed on the skin as consequence of gravity pressure between two hard planes (bony prominences of the person and hard support on which the patient’s body lies), under specific conditions and during prolonged periods of time.^1^ The outcomes of these lesions affect the quality of life of individuals, while increasing hospital costs and health system expenditure.^2^^-^^4^ Although considered preventable, PU/PL/DU affect between 30% and 50% of patients assessed with high risk and constitute between 10% and 50% of all adverse hospital events.^1^^,^^5^^,^^6^ The existence of Clinical Practice Guides (CPG) for their prevention and management with limited recommendations related with weakness of the evidence that supports them, does not facilitate effectively reducing the problem.^7^^-^^10^Among the interventions reported in the CPG that best support effectiveness in preventing PU/PL/DU there are use of anti-decubitus mattresses, use of gels during surgery, and use of protective sponges in the sacral region. Other interventions, such as using dressings, moisturizing creams, use of pressure-reducing pads and scheduled repositioning are carried out empirically with little evidence about their effectiveness.^7^^-^^10^

Postural change, or also called bodily repositioning, is considered a preventive-use intervention in the appearance of these lesions that has been used historically by nursing for nearly 200 years.^11^ This practice has migrated over time with various denominations, like mobilization, positioning, position change, and body rotation, to refer to the same action of release body areas to prevent tissue ischemia. The term repositioning emphasizes on the frequency of change and the term repositioning practice, which is the most up to date, refers to the planning of changes (hourly frequency) conducted by caregivers, bearing in mind the degree of risk individuals may have due to their clinical condition.^12^^-^^16^

The start of position changes must be programmed in patients with high risk of injury, that is, those with a series of determining factors, like the inability to move on their own, with complete dependence on a caregiver to move, or required to remain in bed without moving, for example, those with spinal cord injuries, in hospitalization services, or those in critical state and who are under sedation.^3^ Among the changes reported, there are body rotation from a supine position, laying down [with or without elevation of the head of the bed to 30° or 90° inclination] towards the right lateral or left lateral position, planned for serial periods of time.^11^^,^^17^^-^^19^

Although it is known that not moving patients increases the probability of onset of these lesions in patients at risk, the effectiveness of postural changes with frequency less than or equal to every 2 h is unknown (high frequency) compared with postural changes performed with lower frequency every 4, 6, or 8 h (low frequency) in 24 h, in diminishing the risk of developing a PU/PL/DU.^12^ This article presents a literature review to explore and analyze the current situation of the PU/PL/DU, pathophysiological, epidemiological aspects, and risk factors. Likewise, this review evaluates the progress of the scientific evidence reported on the effectiveness of preventive repositioning on the appearance of PU/PL/DU during the practice of caring for hospitalized vulnerable patients.

## Methods

A narrative review^20^ was structured of the current state of the literature about repositioning as prevention strategy in adult population on the appearance of PU/PL/DU. The search and the bibliographic review was conducted by the very authors, bearing in mind the following attributes: (a) health impact of the PU/PL/DU, description of the physiopathology and the determining factors for the formation of these lesions, (b) repositioning and description of the repositioning interventions applied, (c) immobility and presence of lesion, and (d) impact of repositioning on the prevention of PU/PL/DU. Despite it being a narrative review, the authors report having explored several databases, like the Cochrane Wounds Specialized Register; Medline, Scopus, PubMed, the Cochrane Central Register of Controlled Trials; MEDLINE (Ovid); EMBASE (Ovid), Web of Science, SciELO, and Lilacs, including studies from the last 6-7 years, although for historical data and epidemiological foundation, we include older reviews. The general search terms included [pressure ulcers or pressure lesions, or decubitus ulcers] and [prevention or preventive] and [repositioning or positioning or position changes or postural change] and [adult patient at risk or vulnerable] and [hospitalized or intensive care]. The specific terms with which were combined for the exploration of each objective included incidence or prevalence, physiopathology, risk factors, complications, costs, effectiveness, efficacy, impact. The related articles of preference were systematic literature reviews, randomized clinical trials, observational studies, cost-effectiveness studies, and qualitative studies, in English or Spanish. 

## Results

### Theme 1: Pressure ulcers/pressure lesions/decubitus ulcers: Impact on Health

The PU/PL/DU are currently included among cutaneous ulcers or lesions related with states of dependency and immobility also associated with other concomitant factors, like advanced age, low tissue perfusion, and nutritional alteration.^7^This review kept in mind the terms PU/PL/DU, which are still used to report these lesions globally. The assessment of the rate of PU/PL/DU is considered an indicator of the installed safety and quality of care programs aimed at high-risk patients evaluated in hospitals and health centers.^21^ The high incidence and prevalence of these lesions affects in terms of cost per treatment to patients, society, health services, and assurance systems, which are higher than the costs of health prevention.^21^

Globally, it is estimated that their prevalence in adult population is from 5% to 15% in hospitalized patients, with higher prevalence in intensive care units (ICU) between 15% and 25%.^22^ A systematic review and meta-analysis of global prevalence and incidence of PU/PL/DU, which included cross-sectional and longitudinal studies conducted in hospitals in Asia, Australia, Europe, the Middle East, North America, and South America (surgical, medical and from ICU) between 2008 and 2018, reported calculated pooled prevalence of 12.8% (95% CI 11.8-13.9), a combined incidence rate of 681 885 patients was of 5.4 per 10 000 patient-days (95% CI: 3.4-7.8), and the combined rate of hospital-acquired pressure injuries (HAPI) of 1,893,593 was 8.4% (95% CI: 7.6-9.3%).^23^

More profoundly, another study, the Global Burden of Decubitus study,^24^ presents an epidemiological evaluation by integrating changes in prevalence, incidence, and years of life with disability (YLD) for PU/PL/DU between 1990 and 2019 globally, in hospitalized population. It included for the analysis 369 diseases and lesions, 282 causes of death, and 84 risk factors in 204 countries. The analysis used 7,333 national vital registration systems and 24,657 subnational; 16,984 published studies and 1,654 follow-up household surveys.^25^

The age-standardized rates of prevalence, incidence and years of life with disability (YLD) in 2019 were 11.3 (95% UI 10.2 to 12.5), 41.8 (95% UI 37.8 to 46.2), and 1.7 (95% UI 1.2 to 2.2) per 100-thousand inhabitants, respectively. The study reported a global decrease compared with data from 1990 of 10.6% (95% UI 8.7% to 12.3%) for incidence, 10.2% (8.2% to 11.9%) for prevalence, and 10.4% (8.1% to 12.5%) for YLD (Table 1). Additionally, the global prevalence and incidence rates of the PU/PL/DU increased with age, reaching their maximum point in the age group of 95 years.^25^ The highest prevalence rates per 100-thousand inhabitants standardized by age are reported in the countries included in the high-income region of North America [USA, Canada] (34.6 [31.9 to 37.6]), Latin and Central America [Colombia, Costa Rica, El Salvador, Guatemala, Honduras, Mexico, Nicaragua, Venezuela] (27.4 [24.6 to 30.4]), and Tropical Latin America [Brazil, Paraguay] (24.3 [22.2 to 26.8]).^25^Colombia, despite being among the countries with high prevalence, incidence, and high rate of years of life with disability, is also among the countries that show a decrease in these events. 

**Table 1:** Global changes and in Colombia of Incidence, prevalence, and years of life with disability due to PU/PL/DU between 1990 and 2019^25^*

	1990	1990	2019	2019	
Global and in Colombia	Cases (95% UI)	Rate (95% UI) per 100 000 inhabitants	Counts (95% UI)	Rate (95% UI) per 100 000 inhabitants	Percentage of change in ASR between 1990 and 2019 per 100 000 inhabitants
Global Prevalence	417 024 (375 180 to 462, 07)	12.6 (11.33 to 14.05)	853 854 (776 189 to 942 491)	11.3 (10.19 to 12.48)	-0.1 (-0.12 to -0.09)
Colombian Prevalence	4940 (4483 to 5426)	30.5 (27.4 to 33.68)	15 262 (13 657 to 17 106)	28.1 (25.12 to 31.25)	-0.1 (-0.12 to -0.03)
Global Incidence	1 541 945 (1 389 163 to 1 720 928)	46.5 (41.72 to 52.02)	3 170 796 (2 875 433 to 3 499 729)	41.8 (37.8 to 46.22)	-0.1 (-0.12 to -0.08)
Colombian Incidence	18 268 (16 662 to 20 169)	113.2 (101.61 to 125.72)	57 068 (51 028 to 64 099)	105.1 (94.12 to 118.02)	-0.1 (-0.11 to -0.03)
Global YLD	64 857 (45 376 to 85 486)	1.9 (1.36 to 2.51)	130 238 (92 478 to 171 036)	1.7 (1.2 to 2.2)	-0.1 (-0.12 to -0.08)
Colombian YLD	775 (536 to 1052)	4.6 (3.23 to 6.26)	2328 (1593 to 3147)	4.3 (2.9 to 5.8)	-0.1 (-0.2 to 0.07)

*Information taken from: Zhang X *et al*.,^25^

ASR = Age-standardized rate; YLD = years of life with disability; UI = Uncertainty Interval

Surveys conducted in hospitals in Colombia have reported that the PU/PL/DU of higher prevalence are those Grades I and II (30%), followed by stages III and IV (11%); with greater location in the sacral area (24%), trochanters (19%), glutes (11%), elbows (8%), malleoli and heels (6% each). The most affected patients are those in critical condition hospitalized in internal medicine services (41%), orthopedics (8%), and in ICU services (7%).^26^

The magnitude of these lesions increases health costs generated as consequence of their complications, among which infection is most frequently observed, which can lead to a prolonged hospital stay and death.^3^^-^^5^ Approximate data obtained from several studies have estimated that the total cost of managing a PU/PL/DU, which includes materials, increased bed days, and medications, ranges between $2.2 and $3.6 billion USD/year [Ulcers grade I $12 USD, grade II $373 USD, grade III $3 222 USD, grade IV $66 834 USD).^27^^,^^28^ However, information about the cost per event/patient and annual per countries is very heterogenous, with methodological limitations related with the way of obtaining and analyzing the data. Table 2 shows the data found per countries identified in the literature.^29^^-^^42^

**Table 2:** Approximate cost of PU/PL/DU

Country	Year	Individual cost	Annual/biannual cost	Cost according with grade of lesion
Chile^29^ (USD)	2020	-	Biannual 2018 19 558	-
Iran^30^ (USD)	2019	Grade 1: USD $12 to Grade IV: $66,834	519 991	-
Colombia^31^ (Colombian Pesos)	2018	$84 519	Biannual 369 178 992	-
Canada^32^ (Canadian Dollar)	2017	$26 800 to $233 000 Increased nursing hours: 50%	-	-
Spain^33^ (Euro, €)	2017	-	461 million	-
Singapore^34,35^ (Singapore Dollars)	2016	$4546 to $33 138	-	-
Australia^36^ (Australian Dollars, UD)	2015	$930 million for PU/PL Loss according to bed days: $820 million 525 000 bed days	1.8 billion	Grade I: $747 Grade III: $17 442 Grade IV: $22 467
Belgium ^37^ (Euro, €)	2015	-	4 857 854	Grade I: $67.7 Grade II: $368.4 Grade III: $1,276.3 Grade IV: $2,507.9
New Zeland^38^ (USD)	2015	-	694 million	
USA ^39^ (USD)	2000- 2012	$ 500-$15 200	11.6 billion	Grade I/II: $2770 Grade III/IV: $5630
United Kingdom, UK^40^ (Pound sterling, £)	2012	Per day: £ 43 to 374 (Grade I/II) £ 57 to 343 (Grade III/IV)	3.36 million	Healing cost: Grade I: $1214 Grade II: $5241 Grade III: $9041 Grade IV: $14 108
Germany^41^ (Euro, €)	2012	991 (52/day)	1.3 billion	
Ireland^42^ (Euro, €)	2005	119 000	€ 250 million	-

Regarding prevention, the use of strategies aimed at this purpose has shown a cost-patient savings of up to $2 500 USD and of approximately $7276 USD in the total cost of care.^25^^,^^27^Uncertainty exists about the strategies for effective repositioning practice in reducing lesions, and few studies have reported costs for repositioning (Table 3).^27^^,^^37^^,^^39^^,^^44^^,^^45^

A study about costs related with postural changes to prevent PU/PL/DU reported a repositioning price per minute of 0.58 € (equivalent to $0.63 USD).^28^ Furthermore, it was shown that the time invested in the preventive repositioning of a patient without any of these injuries is less (7.9 min.) than the time invested in the repositioning of patients with PU/PL/DU (10.4 min.). This is equivalent to a cost of 4.6 € ($5.05 USD) for preventive repositioning (without ulcer) and a cost of 6.0 € ($6.58 USD) for repositioning as part of the treatment of patients who have developed an ulcer.^28^^,^^29^ The clinical trial proposed by Moore *et al.*,^44^ compared the incidence of PU/PL/DU and the cost associated with two types of repositioning frequencies at 30 grades° head of bed elevation (30° of inclination tilt). The results showed a lower difference of PU/PL/DU in the group intervened compared with the control group (3% vs. 11%, RR 0.26, 95% CI 0.078-0.90, respectively). The cost per ulcer-free patient was 213.9 € in the group intervened and 283.3 € in the control group. However, these results must be contrasted with other clinical trials of greater magnitude to validate their generalizability.^44^

**Table 3:** Costs of preventive repositioning for PU/PL/DU

Country	Year	Cost Day/patient
USA^39^ (USD)	2019	$867
Australia^27^(Australian Dollars)	2016	$98.90
Belgium ^37^(€)	2015	$7.88 (SD $8.21)
Brazil^45^(Brazilian Real)	2015	$5.38 (SD $6.57) to $8.15(SD $5.8) $31.04 total mean
UK^44^(€)	2013	$287.3 Nursing cost: $25 310

### Theme 2: Pathophysiology and determining factors of their formation

The PU/PL/DU are formed as consequence of ischemic necrosis on the skin and subcutaneous tissue, secondary to increased pressure exerted on any of the bony prominences (such as the sacrum, trochanters, scapulae, heels, and elbows, among others) in immobile patients, elderly adults, and those with greater fragility.^1^ Under normal conditions, maximum capillary pressure is around 20 mmHg and mean tissue pressure varies between 16 and 33 mmHg; however, the presence of pressure higher than these in a given area and exerted for a long time generate the risk of increased ischemic processes that progress up to tissue necrosis.^43^ Subjecting the tissue to greater compression is directly related to diminished blood flow (damage to microcirculation that decreases oxygen delivery), generating ischemia of the vascular membrane, vasoconstriction in the area, with initial erythema characteristic of PU/PL/DU, in addition to extravasation of fluids and cellular infiltration in the area. If said pressure does not diminish and remains so for a sustained time > 2 h, venous thrombosis is generated ending in cell death, necrosis, and ulceration of the tissue.^7^^,^^46^ The different stages of PU/PL/DU are described from slight to severe (I-IV, and other undetermined stages), depending on the degree of involvement in the skin and subcutaneous tissue.^6^^,^^7^

Multiple studies have described the factors associated with the onset of PU/PL/DU. The existence of extrinsic factors is related to the appearance of PU/PL/DU, among these factors are rubbing, friction, increased surface temperature and body humidity or of the area at risk.^47^^-^^51^

The extrinsic factors were reported in the systematic review by Lima-Serrano *et al.,*^47^ which condenses the findings obtained from 17 studies, and the description of the determining factors of PU/PL/DU observed in 19,363 patients hospitalized in different ICUs. The results reported that age ≥ 65 years (OR 2.14, 95% CI 1.27-3.62) and presence of diabetes (OR 5.58, 95% CI 1.58- 18.7) were the two determining factors of higher prevalence of these lesions. In this same study, the factors of higher prevalence related with care were the duration or permanence for long periods of time with an average arterial pressure < 60-70 mmHg (OR 1.09, 95% CI 1.02-1.17), being exposed to mechanical ventilation (OR 23, 95% CI 6.42-86.6), receiving continuous veno-venous hemofiltration treatment, or intermittent dialysis (OR 3.7, 95% CI 1.03-13.86), and having treatment with sedatives (OR 1.02, 95% CI 1.01- 1.03). Likewise, this systematic review identified that in terms of care, performing few postural changes was associated with higher presence of PU/PL/DU (OR 3.63, 95% CI 1.09-12.05).

Regarding postural changes, some studies have reported a higher PU/PL/DU trend in patients who are moved between 4 to 6 times per day (OR 3.63, 95% CI 1.09-12.0), that is, approximately every 4 h in 24 h with a probability of 2.96 for developing Grade II PU/PL/DU (95% CI 1.23-7.15).^48^^-^^50^

### Theme 3: Repositioning in prevention of PU/PL/DU

Postural changes are interventions to prevent PU/PL/DU, which have remained as conventional part of the care patients throughout history. Although it is a valid intervention, it still lacks sufficient evidence to support its effectiveness associated with the frequency of position changes in immobile patients. Postural changes are defined as body repositioning practice regimens performed to redistribute and release the pressure exerted between the body and the support surface upon which the patient is located, including body rotation in lying position, accompanied by elevating the bed angle.^51^^,^^52^ It has been found that the 90° lateralized supine position for more than 2 h decreases blood flow and leads to very low oxygen levels (close to anoxia levels); and positioning patients laterally with a 30° inclination improves transcutaneous oxygen levels, favoring the prevention of these ulcers on the skin and other complications associated with immobility, such as pneumonia, muscle contractures, deconditioning, or urinary tract infections.^52^

Bodily rotation or repositioning strategies may be manual or mechanical; the latter have been implemented with technological progress in health care. Manual body repositioning has traditionally been performed with time intervals (high frequency every 2 h or lower frequency every 4, 6, or 8 h).^51^ However, much uncertainty still exists about which could be the best repositioning frequency with the best benefit for the patient. Current research evidence that mobilization carried out with intervals every 2 h diminishes pressure time over the soft tissue and, likewise, a decrease on the damage generated on the blood capillaries. This practice is performed in distributed and organized manner when the patient is changed from supine position to lateral position (right or left) and back to supine position.^52^ Other elements, such as support surfaces or pads, are helpful in applying this intervention.^7^^,^^12^

Repositioning performed by alternating pressure air surfaces (also called active distribution mattresses) can reduce the incidence of PU/PL/DU compared with foam surfaces, however, the certainty of the evidence is low in terms of cost effectiveness.^53^ Overall, these surfaces allow the release of pressure in specific body zones through automatic and continuous programming, but they partially mobilize the patient in bed. Some of these devices are a combination between specialized beds and lateral repositioning mattresses, which can diminish the work burden of nursing professionals.^53^ Currently, the use of devices that monitor the frequency of repositioning has also been added. These are portable sensors that can be attached to patients in ICU and use artificial intelligence to program mobilization alarms. The use of sensors has shown that, when used, these warn about the moment when a patient must be repositioned, allowing the evaluation of caregivers' adherence to mobilization protocols. In addition, a reduction in the appearance of PU/PL/DU is evident, although said sensors have not yet been fully commercialized in developing countries.^54^

### Theme 4: Relationship between immobility and formation of PU/PL/DU

A relationship exists between the development of PU/PL/DU and loss of independence in spontaneous and autonomous mobilization of individuals. Patients who reposition themselves develop less ulcers, and some studies have observed that a traditional mobilization frequency of every 2-3 h could be an option in reducing these lesions.^18^ However, in the current and routine practice conducted by the nursing staff, this interval has been increasing (lower mobilization frequency per shift), becoming an additional factor for the development of PU/PL/DU.^53^ These failures in reducing the frequency of position changes can be explained by administrative-type factors, related with health costs, like, for example low ratio of nurses per number of patients, limited type and number of beds with specialized mattresses to prevent PU/PL/DU, lack of adequate hospital prevention elements, higher complexity of patients, but above all the existence of limitations in the evidence about the effectiveness of repositioning and prevention interventions.^55^^,^^56^

Some studies suggest that the repositioning interval could be every 3-4 h, using pressure redistribution mattresses, a technology that depends very little on the decision of caregivers and increases hospitalization costs.^6^^,^^55^^,^^56^ However, mobilization frequencies with greater time intervals (3 h, 4-5 h) seem less like the natural (or, one might say, normal) frequency of position changes that an adult individual makes during sleep, state in which individuals move according with their individual needs [at least once/hour during a 7-h sleep period].^56^^-^^58^ This could influence on the intervention, but perhaps more importantly, could also indicate the need for a higher repositioning frequency in patients who cannot make spontaneous movements independently (*e.g.,* patients with spinal cord injury at cervical level or exposed to sedation in the ICU). Although repositioning is often associated with the prevention of PU/PL/DU, it is also important to minimize other types of problems related with prolonged immobility, such as spasticity, muscle rigidity, lack of sensory input, orientation and relationship with the environment, awareness of body image, while minimizing respiratory and vascular complications.^58^

Some factors favor the appearance of these lesions in patients with immobility and dependence on caregivers. The use of medications or treatments with sedatives and analgesics are factors associated with the appearance of PU/PL/DU, given that these reduce sensitivity to pain caused by the prolonged stay in the same position. Further, repositioning may be determined by medical prescription of immobilization in special cases, such as hemodynamic instability, presented by some patients in critical state.^56^In the usual hospital care and ICU practice the repositioning frequency is normally applied in the morning or afternoon shifts, but this mobilization is reduced during the night shifts.^56^^-^^58^ Reduction in the frequency of position changes during the night may be explained by the premise of respecting the circadian sleep cycle, or due to the lack of available care staff, or only due to patient hygiene reasons, considerably increasing the level of risk of injury.^56^

The impact of the frequency of mobilization carried out per shift (day-night) on the appearance of injuries is still unknown, although the description of these factors is based on observations by caregivers, it must be considered when planning the patient repositioning program. 

### Theme 5: Some progress about the impact of repositioning

The limitations on the evidence about repositioning include research related with turning times, body elevation angle, or optimum manual repositioning. An observational study conducted in 1999, the first related with nursing research, analyzed in adult population three turn groups (every 2 to 3 h [n = 32], every 4 h [n = 27], or between 2 and 4 times per day [n = 41]), observing that elderly adults who were repositioned every 2 to 3 h had less ulcers.^59^ This historical study created the gold standard that would support the practice of mobilization every 2 h in vulnerable patients. Conversely, a study carried out some years later by DeFloor *et al*., suggested that depending on the support surface used (for example, viscoelastic surface in mattresses), less frequent turning could be optimal to prevent PU/PL/DU in patients hospitalized in long-term care facilities.^60^ However, these findings have been questioned by other authors, who have suggested that it may be too soon to abandon the mobilization routine of every 2 h in favor of every 4 h based on this study.^61^^,^^62^

The systematic review by Gillespie *et al*.,^63^ to assess the clinical and cost-effectiveness aspects of repositioning to prevent PU/PL/DU in adult population, included eight clinical trials conducted in diverse hospitalization services. It analyzed 3,941 patients included in the clinical trials evaluated; however, only three clinical trials (including 1,074 patients) compared the 2-h repositioning frequency against every 4 h on the onset of PU/PL/DU. The results showed non-significant differences between both interventions (Relative risk 1.06, 95% CI 0.80 to 1.41, I^2^ 45%). This review also identified two studies (with 252 participants) that compared two types of bed repositioning (30 degrees versus 90 degrees of inclination) in the reduction of PU/PL/DU. The results showed no significant differences between both interventions (Relative risk 0.62, 95% CI 0.10 to 3.97, I^2^ 69%). 

## Discussion

The literature synthesis provides a global sample of the current state of PU/PL/DU developed by adults during hospitalization; a health situation that continues being of great magnitude and which affects patients, their families, society, and health systems in general. Furthermore, a review is presented of the pathophysiological aspects and determining factors that lead to their formation. Finally, a prevention intervention is explored, like repositioning or position changes in preventing these lesions and the scope of the evidence in terms of effectiveness at hospital level. 

Among the most-relevant epidemiological aspects of this review there is the existence of data that show a global reduction of the prevalence and incidence and of the YLD between 1990 and 2019.^24^^,^^25^ Despite these results, Colombia still reports high PU/PL/DU rates, aspects that must be disclosed and taken into account to implement better control measures of events and of hospital prevention, with a multiple approach.^6^^,^^26^

The magnitude of these lesions increases health costs around 1.4% - 1.9% of public spending, which are generated as a consequence of their complications, mainly infections that can lead to increased hospital stay and increased mortality.^27^^,^^28^ However, obtaining this information is affected by the scarce number of cost-effectiveness studies conducted in that regard and by the great heterogeneity in the methods reported. In addition, the detailed costs presented by some studies include high-cost prevention strategies, such as the use of dressings (hydrocolloid), among others, with scant evidence reported of their effectiveness.^24^^,^^27^^,^^39^^,^^40^^)^

Repositioning, or body rotation of individuals in state of total fragility and dependence at hospital level, is a care intervention used historically and empirically to prevent PU/PL/DU. Position changes could prevent the appearance of these lesions, avoiding the development of the more complicated and costly injuries to treat: grades III and IV PU/PL/DU.^63^Although the scope of the evidence about this intervention’s effectiveness is quite weak, it is known that the release of pressure on body areas contributes to better tissue perfusion, considering that these lesions can develop in less than 2 h remaining in the same position. However, to date, high-quality evidence has not been observed to permit supporting or modifying this practice. 

Moreover, it is necessary to keep in mind the challenges implied to the nursing staff by using these types of interventions and which may be determinant for their application, like - for example, sufficient number of staff to carry out the rotations, support systems to control time, and use of devices to facilitate displacement during the turns.^55^ Additionally, the particular conditions of the patients’ diseases could also be limitations to apply these types of interventions. 

Including weak evidence in CPG based on studies of low methodological quality delays progress in research related to these types of interventions. Some limitations identified within the studies available are related with the lack of blinding mainly of those who evaluated the outcomes, failures in reporting (or non-performance) of randomization, and lack of precision of the results, the context of where these took place (mostly in developed countries and ICU) that diminishes the possibility of generalizing their results to other scenarios. In turn, most of the studies had a small sample size, which limits their power and leads to over or underestimation of results.^62^^,^^64^

In that sense, uncertainty persists regarding the adequate type and frequency for repositioning in hospitalized vulnerable patients. Currently, the nursing care plan for hospitalized patients includes position changes and its frequency lies at the discretion of each professional, bearing in mind the analysis of the multiple factors that determine the feasibility of its use. It is also not clear if the repositioning frequency should be modified during the night period considering that thus there is less spontaneous movement from the patients and, thereby, greater body pressure. Consequently, further research is needed with more-robust methodological approaches that permit obtaining valid results that guide appropriately the clinical practice. 

The PENFUP phase 2 research project is a pragmatic randomized controlled clinical trial in 22 clusters [hospitals], led by nursing and funded by MINCIENCIAS in Colombia, which is finishing its development (Registry in Clinical Trials.gov NCT04604665). This project sought to assess the effectiveness of two levels of manual repositioning to prevent PU/PL/DU acquired in adult patients hospitalized in various ICUs in different Colombian cities. Being a cluster randomized clinical trial, the ICUs of the hospitals will be randomized to a high-frequency repositioning (position changes between 1-2 h of eligible patients plus reinforcement of care via telegram and SMS text message to the caregivers) or to a conventionally administered frequency repositioning, thus, allowing to evaluate the primary outcome: incidence or onset of new PU/PL/DU by ICU groups.^65^ Although this study is conducted particularly in critical care settings, it is expected for its results to contribute to solving the knowledge gap related with the repositioning practice not only in this population, but to provide guidelines for the management of other vulnerable patients. 

## Conclusions

The narrative review performed provides valuable information about the epidemiology, costs, and the state of the research related with repositioning, a prevention intervention used for over 200 years to control the onset of PU/PL/DU in vulnerable adults during hospitalization. The literature reports that, although a decrease exists in the incidence, prevalence, and years of life due to disability due to these lesions globally between 1990 and 2019, the rate of events is still high and continues producing a high social and economic impact on health that must be mitigated. Among the interventions applied to prevent these events, there is the repositioning of patients; an intervention that is carried out empirically during patient care. Progress in research on the effectiveness of repositioning to prevent PU/PL/DU shows no differences in reducing these lesions when comparing different strategies and frequencies in position changing of patients at risk of injury. High-quality research must be promoted to determine the effectiveness and costs related with implementing preventive repositioning that leads to improving the clinical results of patients at hospital level.
